# The relationship between management practices and the efficiency and quality of voluntary medical male circumcision services in four African countries

**DOI:** 10.1371/journal.pone.0222180

**Published:** 2019-10-03

**Authors:** Andrea Salas-Ortiz, Gina La Hera-Fuentes, Nerissa Nance, Sandra G. Sosa-Rubí, Sergio Bautista-Arredondo

**Affiliations:** 1 Division of Health Economics and Health Systems Innovations, National Institute of Public Health (INSP), Cuernavaca, Mexico; 2 University of York, York, United Kingdom; 3 University of Newcastle, Newcastle, Australia; 4 School of Public Health, University of California, Berkeley, California, United States of America; University of the Witwatersrand, SOUTH AFRICA

## Abstract

**Introduction:**

Given constrained funding for Human Immunodeficiency Virus (HIV) programs across Sub-Saharan Africa, delivering services efficiently is paramount. Voluntary medical male circumcision (VMMC) is a key intervention that can substantially reduce heterosexual transmission—the primary mode of transmission across the continent. There is limited research, however, on what factors may contribute to the efficient and high-quality execution of such programs.

**Methods:**

We analyzed a multi-country, multi-stage random sample of 108 health facilities providing VMMC services in sub-Saharan Africa in 2012 and 2013. The survey collected information on inputs, outputs, process quality and management practices from facilities providing VMMC services. We analyzed the relationship between management practices, quality (measured through provider vignettes) and efficiency (estimated through data envelopment analysis) using Generalized Linear Models and Mixed-effects Models. Applying multivariate regression models, we assessed the relationship between management indices and efficiency and quality of VMMC services.

**Results:**

Across countries, both efficiency and quality varied widely. After adjusting for type of facility, country and scale, performance-base funding was negatively correlated with efficiency -0.156 (p < 0.05). In our analysis, we did not find any significant relationships between quality and management practices.

**Conclusions:**

No significant relationship was found between process quality and management practices across 108 VMMC facilities. This study is the first to analyze the potential relationships between management and service quality and efficiency among a sample of VMMC health facilities in sub-Saharan Africa and can potentially inform policy-relevant hypotheses to later test through prospective experimental studies.

## Introduction

Despite significant progress in the past decade[1], HIV remains a global challenge. At the end of 2015, there was a total of 36.7 million people living with HIV.[2] The burden is concentrated most heavily in sub-Saharan Africa, where 71% of people living with HIV reside.[1] This regional epidemic is primarily explained by heterosexual transmission.[1] Among available strategies to reduce the spread of HIV, male circumcision has been shown to reduce the rates of female-to-male sexual transmission by 60%. [3–5] Not only is VMMC critical to the future reduction of the burden of HIV, but it also can aid in reducing the transmission of other sexually transmitted diseases like human papilloma virus. [6] Consequently, the procedure has been prioritized for scale-up in the sub-Saharan African region.[7] To date, however, scale-up targets have only been partially met.[7]

In order to meet these targets in the future given current financial constraints, one important aspect to examine is the role of service delivery efficiency. Through higher efficiency in the implementation of VMMC services, coverage targets could be met, new HIV cases could be reduced, and long-term HIV-related costs could decrease.[8–11] However, while achieving higher efficiency is imperative, it remains unclear to what extent the focus on the economics of VMMC delivery alone may cause programs to neglect aspects related to quality of services. Studying quality and efficiency in tandem is one way to address this concern; however, few studies have done so for HIV programs. Among those that have, a recent study in Kenya and Swaziland found that integration of HIV and sexual reproductive health services led to an increase in both efficiency and quality of services. [12] For VMMC in particular, a Tanzanian study found that a campaign to scale up VMMC led to more efficient and higher quality services. [13].

These studies, however, are limited in geographic scope and do not explore determinants of efficiency and quality, which remains an unexamined question across low- and middle-income country contexts.

One aspect of service delivery that could ensure both efficiency and quality is facility-level management practices. Management can affect the provision of services through a variety of pathways. Staff management may stimulate performance and improve quality through a combination of positive incentives (bonuses, financial rewards, preferred rotation, time off) and sanctions (penalties, warnings to health staff). [14] In 2010, the World Health Organization (WHO) released a manual for optimizing the volume and efficieny of VMMC services. These guidelines entailed different considerations related to the use of personnel time. For instance: task-shifting and task-sharing practices; optimization of the use of facility space, client flow and scheduling, and supply chain management. [15] Management may also catalyze good performance by optimizing processes through supply chain management and financial planning.[16] To this point, some studies have found a link between management practices and positive clinical and financial outcomes. [17,18] Yet efforts in recent years to understand costs and cost drivers for VMMC services [9–11,19] have not addressed the potential role of management in explaining differences in efficiency and quality. In the analysis presented in this paper, we leverage a multi-country study measuring efficiency, quality and management practices in a sample of 108 facilities that supply VMMC services. Our objectives are to explore the heterogeneity of quality and efficiency of VMMC services across countries and to examine the ways in which management practices interact with both the efficiency and quality of VMMC services.

### The economics of management

**Fig 1:**
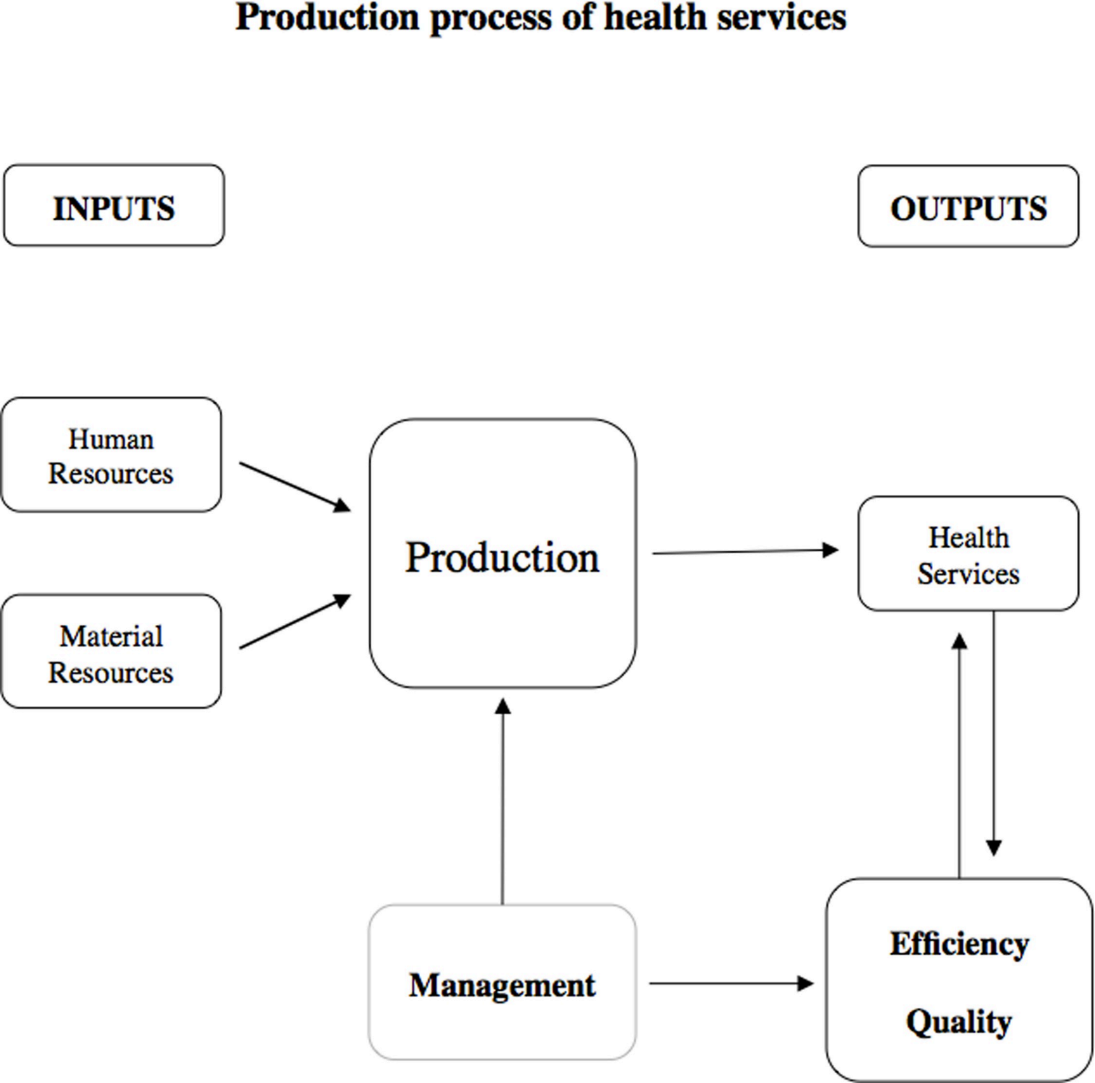
Management, efficiency and quality in the production of health services.

Through a microeconomic lens, a health facility is analyzed as a firm, which uses inputs (human and material resources) to produce outputs (health services) within a production process (Fig 1). These *firms* make decisions regarding how to combine and transform inputs to produce health services, given the technology at their disposal. The efficiency and quality of service delivery can thus be shaped by a clinic’s decision-making.

To this point, economists Van Reenen and Bloom have examined the role of management practices in the production process. Their research has gone far to document how differences in productivity among firms—industrial firms or hospitals—might be explained by management practices. [18,20] This research was later expanded to include public hospitals, where there is less competition and therefore less incentive to be efficient.[17].

While ‘management’ can be complex and difficult to define and measure, some specific management practices have been shown to be relevant in the health sector, and particularly in the HIV field. Not only can management practices affect productivity, they can also alter key aspects of healthcare delivery like efficiency and quality. Human resource management and community participation are examples of this. In 2010, Bloom and Van Reenen found that some human resource management practices increased US firm productivity. These results may also translate to the health sector; efficient and high quality provision of services depend greatly on proper organization, direction and motivation of the health personnel. Conversely, poor management of personnel can lead to superfluous tasks, duplication of activities, staff demotivation and waste of resources—increasing inefficiencies and depleting quality. Additionally, management practices that favor community engagement can increase service access, social cohesion and capital. We utilize the theoretical framework built from this literature to explore how management practices can affect the quality and efficiency of HIV service delivery.

## Methods

### Data collection

Cross-sectional data for this project were collected as part of the “Optimizing the Response in Prevention: HIV Efficiency in Africa” (ORPHEA) study, conducted between October 2012 and December 2013 in Kenya, Rwanda, South Africa and Zambia. [21] Standardized study instruments were designed to measure management practices, *inputs* (personnel, recurring supplies, utilities and capital) and *outputs* for HIV Counselling and Testing (HCT), Prevention of mother-to-child transmission (PMTCT) and VMMC services (number of HIV tests; women enrolled in prevention of mother-to-child transmission services, and number of circumcisions performed). For each country sample, a multistage sampling strategy was used to select subnational areas.

A sampling frame was constructed, including sub-national areas, these were selected purposively based on local HIV prevalence and other logistical considerations, namely safety and presence of the provision of HCT, PMTCT and VMMC services. The facilities in these subnational areas were stratified by the facility ownership (public/private) and the level of service provision (primary, secondary, tertiary). Within each stratum, health facilities were randomly selected using probability proportional to the total annual number of patients served in outpatient services. Preference was given to integrated sites (defined as those providing the three interventions—HCT and PMTCT and VMMC), rather than stand-alone facilities (defined as those that only provide one type of intervention). Integrated sites were included in the sample with probability of one if the number of integrated sites was less than the sample size, which was the case for all countries. The final sample included both integrated and stand-alone sites.

In total, there were 23 sub-national areas included in the sample, distributed as follows: in Kenya, 10 out of 47 counties; in Rwanda, all 5 provinces; in South Africa, 3 out of 9 provinces; and in Zambia, 5 provinces out of 9. Further details about the sample strategy are explained elsewhere. [21] The survey applied a micro-costing approach, which combined the collection of primary data on inputs, input prices and outputs with direct observation of time allocation of providers to specific tasks and services to estimate the total economic costs of production of VMMC.

The instruments collected retrospective monthly data and were designed to capture information from program records. Input data were obtained independently of funding source; donated inputs were valued using local market prices and output data (total number of procedures provided) were collected monthly from program records. For the purposes of this analysis, we included only facilities that provided VMMC services. Our final analytical sample consisted of 108 facilities in Kenya (N = 33), Rwanda (N = 32), South Africa (N = 26) and Zambia (N = 17).

### Measurement

#### Efficiency

To calculate efficiency, we estimated facility-level efficiency scores using Data Envelopment Analysis (DEA). DEA is a non-parametric, linear programming method that uses observed combinations of inputs and outputs to estimate an empirical production frontier. [22] The technique evaluates the extent to which the observed performance of a decision-making unit (DMU; in this case, each facility), reaches an empirically ‘optimal’ level. This optimal level is defined by the best practices (production function) frontier, *i*.*e*. the results attained by the observed DMUs with the most efficient performance. After identifying the best practices frontier, DEA computes the distance from the efficiency frontier to each DMU, providing individual measurements of relative efficiency (efficiency scores) and subsequently ranking DMUs in terms of efficiency. As DEA is a non-parametric method, it does not require any assumption of a specific functional form describing the production process.[22,23] Based on prior literature, we estimated efficiency scores assuming an output-oriented approach, and a model with variable returns to scale. [24–27] Our output-oriented approach assumes that health facilities tend to have more control over the outputs rather than inputs. [12] This is particularly appropriate for the African context in public facilities, where most HIV programs tend to be managed centrally, health facilities have limited control over the designation of resources, and health labor markets are relatively inflexible. We assumed variable returns to scale, which allows for both increasing and decreasing returns to scale. We found this to be most appropriate because of the shape of the VMMC cost function. There were high unit costs per VMMC at low levels of services supplied. As the number of services increased, the mean cost fell sharply (*i*.*e*. economies of scale), but once a certain level was reached, the average cost started to rise—or at least stop decreasing—as more services were added (*i*.*e*. diseconomies of scale). [12]

Facility-level average costs or unit costs were defined as the health facilities’ total annual input costs divided by the annual number of interventions (outputs) performed, as follows:

$$Unit\;cost\;VMMC_{i}=\;\frac{\sum_{c=1}^{c=n}I_{ci}}{Total\;VMMC\;performed_{i}}$$

where the term *I* represents the input category *c* for the *i* facility. Variables related to capital, utilities, and staff were specified as inputs, while the number of VMMC clients was considered as the output (S1 Table). The costing year of this analysis is 2013. DEA scores range from 0 (least efficient) to 1 (most efficient). DEA scores were multiplied by 100 to ease the interpretation and analysis.

#### Quality

Quality scores were obtained through a vignette questionnaire that presented a clinical case. The structure and content of the instrument was designed based on available national guidelines in each country, as well as WHO guidelines, at the moment of data collection (S2 Table). [21] This instrument aimed to capture provider’s competence and compliance to the guidelines. [28] The questionnaire evaluated 74 items, including pre- and post-VMMC procedures and post-operative complications. Up to five providers per facility answered the clinical vignettes; respondents included doctors, nurses, and other health staff. [21] Interviewers applied the survey by describing a hypothetical case (a 26-year-old HIV-negative man who arrives to the clinic seeking VMMC services) and then asking what the provider would do during the MC procedure. The interviewer then recorded the extent to which the unprompted reply coincided with guidelines, which entails pre-MC evaluation, counselling and post-surgical procedures and counselling. The interviewer recorded answers spontaneously mentioned by the interviewee (they did not read out answer options). A score from 0 to 100 was assigned to each provider based on the proportion of correct answers they mentioned out of the total possible answers. The higher the score, higher the quality. The scores showed high internal consistency (Cronbach’s alpha α = 0.94), meaning the questions consistently measured the same concept across all items.[29] Quality scores per facility were calculated by collapsing scores at the mean.

### Management

Based on our theoretical framework, we constructed six indices to serve as proxy measures for management practices at the facility level. The questionnaire was applied as part of the facility-level survey. In a face-to-face interview, enumerators asked facility managers questions on management practices implemented at the facility, if needed data was abstracted from records (accounting records, staff payroll, performance reports & records, human resource records and inventories). However, many of the answers in this section rely on self-reported information. The questions were adapted from the literature and to the context of the four countries studied. [17,18,30] We focused on the following six dimensions of management: performance-based funding, sanctions, external supervision, community participation, national-level governance and municipal-level governance. These dimensions capture a breadth of management practices that can affect clinic performance, and were informed by the literature and a previous analysis of the data. [31] All indices were standardized to a range from 0 to 100, scores closer to 100 show better management practices.

The *performance-based funding* index details the incentives to encourage individual- or clinic-level performance. The *sanctions* index measures the types of sanctions linked to poor performance, applied at the provider level. Both incentives and sanctions can extrinsically motivate better staff performance, and therefore improve facility-wide results. It is not clear, however, how these mechanisms relate to costs and quality. The *external supervision* index measures the amount of oversight higher-level facilities have over the clinic. The *national-level* and *municipal-level governance* indices measure the amount of involvement that national and municipal governments, respectively, have over decisions at the facility level—specifically, on budgets and expenditures. More oversight may lead to improved efficiency, but alternatively, it may produce burdensome and costly administrative work.

S3 Table in supplemental materials describes the specific questions used to measure each dimension of management. For each category, we constructed an index using additive scores; their internal consistency was validated using Cronbach’s alpha, a measure of internal consistency. A high Cronbach’s alpha reflects a high average inter-item correlation. A score higher than 0.7 is considered as “acceptable” [31]. In our analysis, Cronbach’s alpha were higher than 0.80.

We further validated the management indices by applying Principal Component Analysis to all variables used in the six scores. Principal components analysis is a data reduction technique which creates “scores” that are linear combinations of a group of variables. Each score has variables that “contribute” more than others (i.e. have a higher positive magnitude). We found that when we looked at the variables that most contribute to each score, the resulting groupings across the PCA scores were similar to those in our indices. This lends further evidence that score components, and therefore management categories, are internally consistent.

#### Efficiency-quality quadrants

We categorized facilities into four groups based on the correlation of the efficiency and the quality indices, using the median values of the distribution of each score as cutoff points. Quadrant I includes “high performance” facilities, i.e. facilities with both quality and efficiency values above the median, while quadrant III includes “low-performance” facilities with values below the median.

#### Additional facility-level characteristics

Other characteristics included in the analysis were country and service provision level (S4 Table). The variable type of facility has two categories: hospitals (reference category) and primary care clinics.

### Statistical analysis

To examine the relationship between management practices and efficiency and quality scores, we first described summary statistics and the dispersion of facility characteristics, and the quality and efficiency scores by country. We estimated the relationship between management, quality and efficiency using generalized linear models (GLM). As health cost data tend to be skewed, GLM offer a greater flexibility over linear models: relaxing the assumption of normality and allowing for a non-uniform variance. Contrary to Ordinary Least Squares (OLS), where ther error term is independent and identically distributed, GLM allow the variance not to be constant for all observations, but a function of the mean. Then, to determine the distribution family used with GLM, we applied the Modified Park Test[32] and we used the Gaussian family specification.

Then, to take into account the hierarchy of the data and the differences across countries, we also estimated mixed-effects (ME) models. The rationale behind the use of theses models is the fact that they are more flexible in the estimation of country-level effects. Mixed-effects models allow the coefficients to vary across countries by estimating the between-country variance and covariance. Thus, the natural heterogeneity in the data of the different countries is accounted. To test the goodness of fit of these models, we carried out the Likelihood Ratio Test (LRT), which tests whether random effects are equal to zero. Since the test was significant (p<0.05), we included the ME models. Only for the model for quality of services, the test showed that mixed effects models offered a better fit compared with a standard linear regression (p>0.10).

The general specification of the model on efficiency is displayed in Eq I, as follows:

$$E_{i}=\beta_{0}+\beta_{1}x_{1}+\beta_{2}x_{2}+\ldots +\beta_{6}x_{6}+\beta_{7}x_{7}+\beta_{8}x_{8}+\beta_{9}x_{9}+\varepsilon_{i}$$ (I)

The model analyzes the relationship between management and efficiency and includes six variables (x_1_…x_6_) that indicate different management indices (performance-based funding, sanctions, external supervision, community participation, national-level governance and municipal-level governance), and variables measuring volume of outpatients (centered at the mean; x_7_), type of facility (x_8_), and country (x_9_).

Eq II represents the model on quality, in which, as in the case of efficiency, we included management scores (x_1_ to x_6_), adjusted by volume of patients, type of facility (x_8_) and the country variable (x_9_). In both models $\varepsilon_{i}$ represents the error term.

$$Q_{i}=\beta_{0}+\beta_{1}x_{1}+\beta_{2}x_{2}+\ldots +\beta_{6}x_{6}+\beta_{7}x_{7}+\beta_{8}x_{8}+\beta_{9}x_{9}+\varepsilon_{i}$$ (II)

### Efficiency and quality analysis by country

Despite sample size limitations, we also estimated models per each country in order to explore whether there were common patterns in the associations between management variables and efficiency and quality across countries. Country-level models follow the specifications described above.

### Ethical clearance

Written consent was obtained from each facility officer in-charge at the time of data collection. The study and all accompanying materials, including consent forms, were approved by the ethical review boards of several institutions, including: The National Institute of Public Health, Mexico; Kenyatta National Hospital, University of Nairobi, Kenya; Northeastern University, United States of America, (USA); Rwanda Biomedical Center, Rwanda; University of Witwatersrand, South Africa; and University of Zambia, Zambia.

## Results

### Sample

Overall, the mean number of VMMC clients per facility per year was 842. We performed an analysis of variance to examine whether the means were equal in each of the variables and across countries. (Table 1). There were statistically significant differences in number of clients per year between all countries (p<0.05), except for Zambia and Rwanda. Facilities were relatively smaller, on average, in Rwanda (342 clients), and larger in South Africa (1,665 clients). The proportion of primary care facilities was higher in Rwanda (78%), and lower in South Africa (35%). Different combinations of personnel were observed across countries. Facilities in Kenya and South Africa had the highest percentage of medical doctors (24% and 15%, respectively), while clinics in Rwanda and Zambia had the highest percentage of specialized nurses (48% and 41%, respectively). General nurses were most common in South Africa and Rwanda (35% and 29%, respectively).

**Table 1:** Description of facilities included in the sample in terms of clients, type of facility and percentages of staff categories, by country.

Facility-level characteristics^1^	All sample			Kenya			Rwanda			South Africa			Zambia		
	N	Mean	SD^3^	N	Mean	SD	N	Mean	SD	N	Mean	SD	N	Mean	SD
VMMC Clients^**1**^	108	842	1,379	33	869	798	32	342	392	26	1,665	2,415	17	470	533
Percentage of primary health care facilities^**1**^	108	63	-	33	67	-	32	78	-	26	35	-	17	71	-
Percentage^2^ of medical doctors^**1**^	108	13	21	33	24	27	32	2	10	26	15	21	17	8	15
Percentage^2^ of general nurses^**1**^	108	27	28	33	26	28	32	29	34	26	35	23	17	11	17
Percentage^2^ of specialized nurses^**1**^	108	27	34	33	3	14	32	48	36	26	20	27	17	41	36
Percentage^2^ of other health staff ^**1**^	108	20	24	33	30	25	32	9	18	26	19	25	17	25	24

^1^At least one country is statistically different. Analysis of variance (ANOVA) (p<0.05).

^2^ Percentage of staff working on a full-time basis (FTE) out of total staf.

^3^ Standard Deviation (SD).

### Efficiency and quality scores

**Fig 2:**
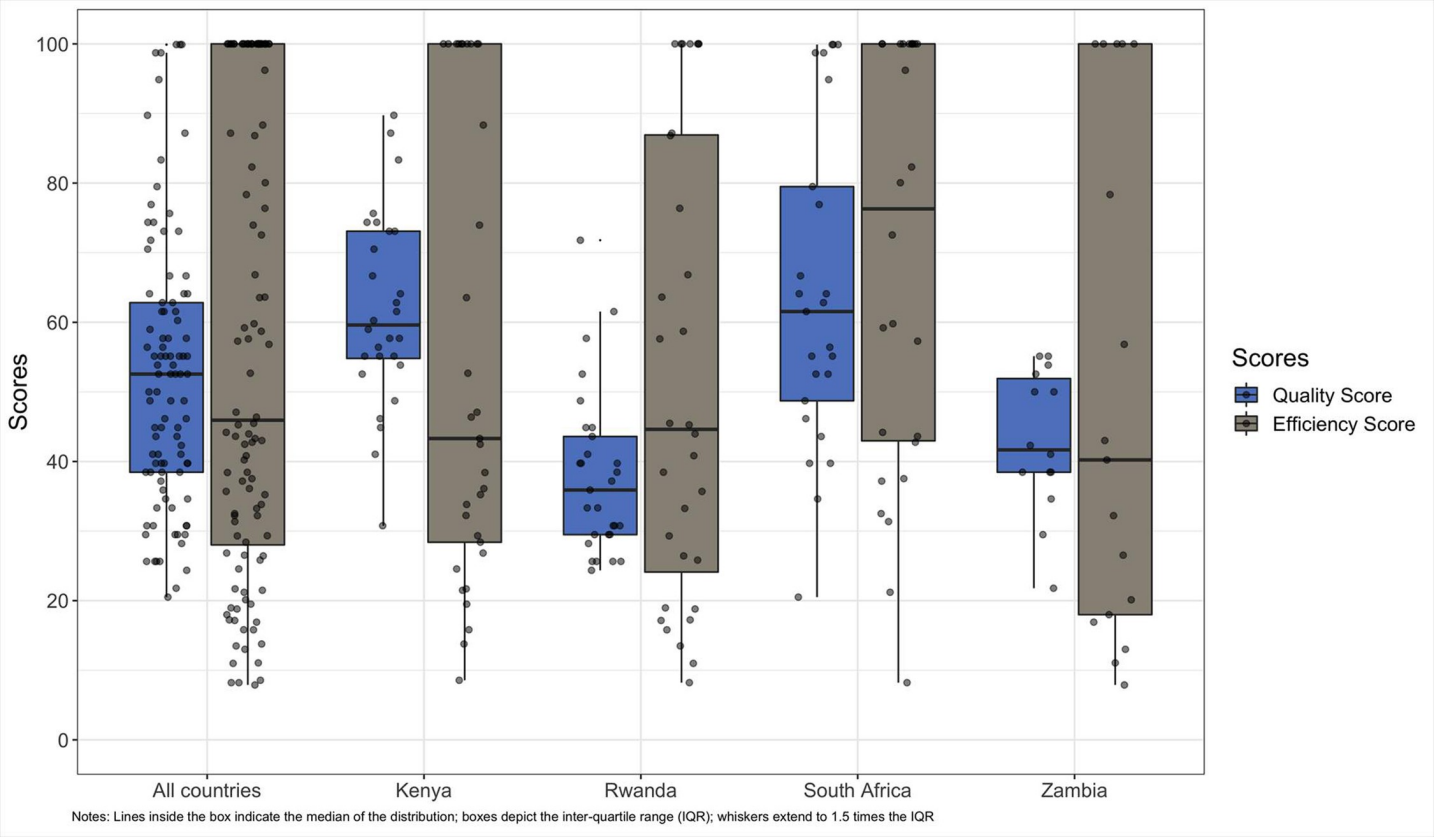
Distribution of efficiency and quality score by country. Notes: Lines inside the box indicate the median of the distribution; boxes depict the inter-quartile range (IQR); whiskers extend to 1.5 times the IQR.

There was significant variation in the levels of efficiency and quality within and across countries (Fig 2 and Table 2). The mean efficiency score ranged from 51 in Zambia to 69 in South Africa. Regarding the quality scores, Kenyan and South African facilities scored mean quality values of 62 and 64, respectively; Rwandan and Zambian facilities, in contrast, had mean values of 38 and 43, respectively (Table 2).

**Table 2:** Efficiency and quality scores by country.

Scores	All sample			Kenya			Rwanda			South Africa			Zambia		
	N	Mean	SD	N	Mean	SD	N	Mean	SD	N	Mean	SD	N	Mean	SD
**Efficiency**^**1**^	108	57	33	33	56	34	32	53	33	26	69	31	17	51	37
**Quality**^**2**^	96	53	20	28	62	14	29	38	12	25	64	23	14	43	10

^1^Efficiency score: With the exceptions of the differences between Kenya and Rwanda, and Kenya and Zambia, all other pair-wise comparisons showed.

statistically significant differences in scores (p<0.05).

^2^Quality scores: With the exceptions of the differences between Kenya and South Africa, and Rwanda and Zambia, all other pair-wise comparisons showed. statistically significant differences in scores (p<0.05).

Note: twelve observations with missing values on the Quality score.

### Management

**Fig 3:**
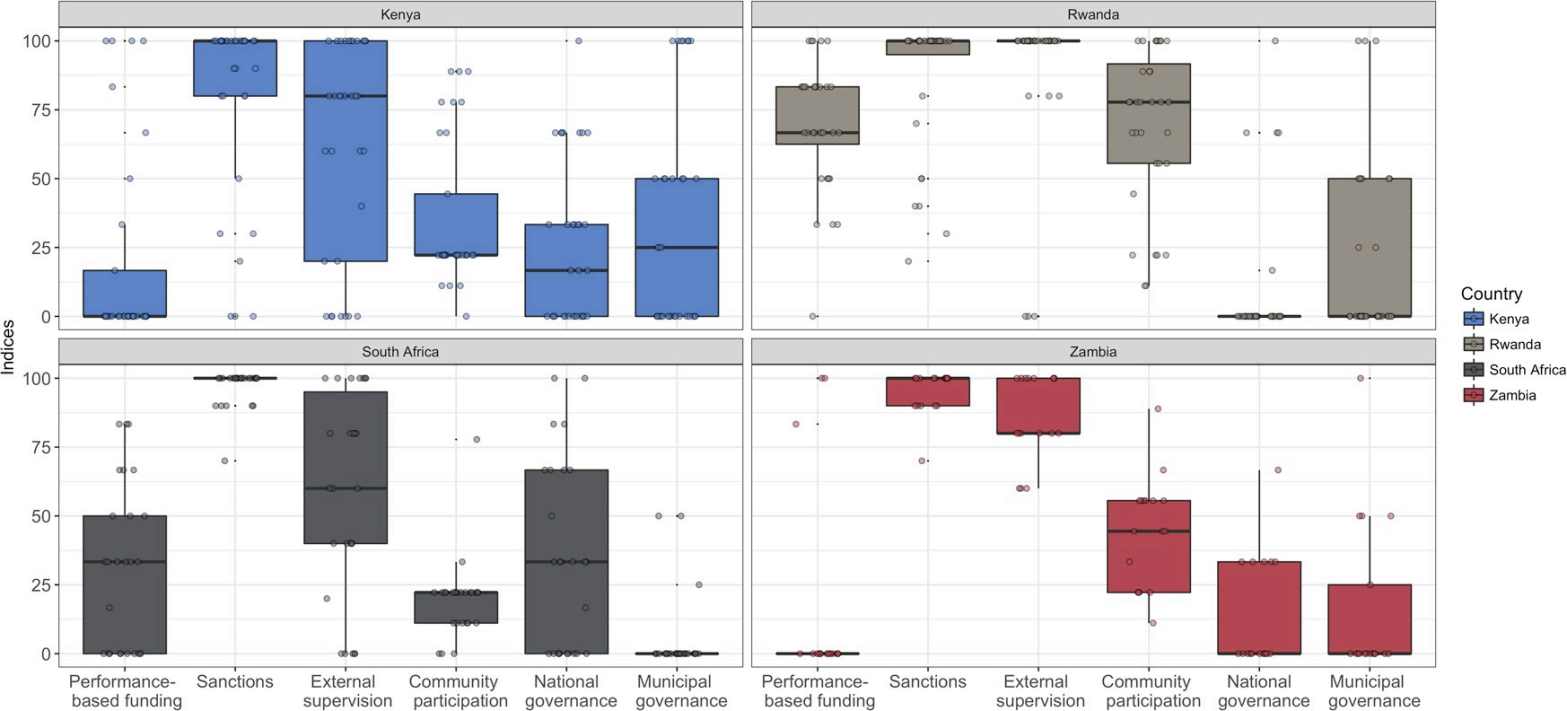
Management scores by country.

We found variability in management practices both within and across countries, as well as across management dimensions (Table 3 and Fig 3). The highest mean performance-based funding score was observed in Rwanda (70). Sanctions were widely implemented in all four countries, with relatively high scores across countries. The lowest sanctions score was observed in Kenya (79). External supervision and community participation scores were highest in Rwanda and Zambia (89 and 86, and 69 and 44, respectively). Involvement of national and municipal authorities in decision-making was relatively low in all four countries. South African facilities on average had the highest national governance score (35) and Kenyan facilities on average had the highest municipal governance score (34).

**Table 3:** Description of the management scores.

Management Scores	All sample			Kenya			Rwanda			South Africa			Zambia		
	N	Mean	SD	N	Mean	SD	N	Mean	SD	N	Mean	SD	N	Mean	SD
Performance-based funding^1^	108	37	38	33	20	36	32	70	23	26	31	30	17	17	37
Sanctions^1^	108	88	24	33	79	34	32	87	25	26	97	7	17	95	8
External supervision^1^	108	73	36	33	62	39	32	89	30	26	58	37	17	86	15
Community participation^1^	108	43	30	33	35	26	32	69	29	26	20	14	17	44	20
National governance^1^	108	21	30	33	26	29	32	10	26	26	35	35	17	14	21
Municipal governance^1^	108	20	33	33	34	40	32	20	33	26	5	14	17	16	29

^1^At least one country is statistically different. Multivariate mean test (p<0.05).

### Quadrants of performance

**Fig 4:**
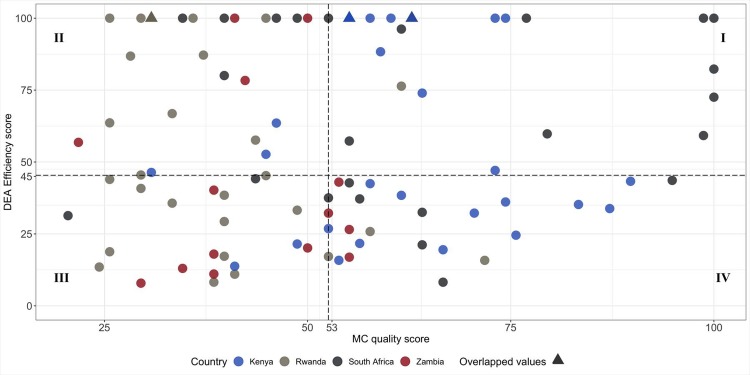
Distribution of facilities by quadrants.

Quadrants I and III in Fig 4 (above and below median efficiency and quality) are comprised of 30 and 29 facilities, respectively. We found some differences across countries (Fig 4). Of the 25 South African facilities, 20 were categorized as high efficiency, though these facilities displayed heterogeneous levels of quality. The same was true for Kenya; most facilities (17 of 28) were considered highly efficient, but these facilities were evenly split between high and low quality (16 and 1 facilities, respectively). In contrast, most Zambian facilities were below the median of the efficiency score (12 of 14) and scored low on quality (8 facilities). The majority of facilities in Rwanda fell into low-quality quadrants (II and III; 25 out of 29 facilities; Fig 4).

*Twelve missing values on the quality score: 3 in Zambia, 1 in South Africa, 3 in Rwanda and 5 in Kenya

### Associations between management and efficiency, and management and quality of VMMC services

In specification I, we examined the efficiency score as the dependent variable, management scores as independent variables, and country, type of facility and volume of outpatients as control variables. We found a negative correlation between performance-based funding and efficiency scores. Volume of outpatients and type of facility showed a significant positive relationship with the efficiency scores (Table 4).

**Table 4:** Association between management scores and efficiency and quality of HIV service delivery.

Management Variables	I: Efficiency	II: Quality	III: Quality
	GLM	GLM	ME
Performance-based funding	-0.17+	0.08	0.06
	(-0.37–0.02)	(-0.02–0.18)	(-0.03–0.16)
Sanctions	-0.04	-0.1	-0.1
	(-0.31–0.22)	(-0.24–0.04)	(-0.24–0.03)
External supervision	-0.08	0.05	0.03
	(-0.26–0.1)	(-0.05–0.15)	(-0.06–0.13)
Community participation	0.19	-0.06	-0.09
	(-0.07–0.45)	(-0.20–0.08)	(-0.22–0.04)
National governance	0.07	0.09	0.1+
	(-0.16–0.29)	(-0.03–0.21)	(-0.01–0.22)
Municipal governance	0.09	-0.05	-0.05
	(-0.11–0.30)	(-0.17–0.06)	(-0.17–0.05)
Non-Hospital	15*	2.6	2.7
	(1.4–28)	(-4.8–10)	(-4.3–9.8)
Outpatients (100s)	0.04**	0.01	0.01
	(0.01–0.07)	(-0.01–0.02)	(-0.01–0.02)
Rwanda	0.86	-26***	
	(-20–22)	(-37–-15)	
South Africa	6.5	-2.58	
	(-17–30)	(-15–10)	
Zambia	-8.0	-18**	
	(-28–12)	(-29–-7)	
Observations	108	96	96
Number of Groups			4

Note: Kenya is the country of reference. GLM (Generalized Linear Models- Gaussian family), ME (Mixed-effects Models) 95% Confidence interval in parentheses.

*** = p<0.001.

** = p<0.01.

* = p<0.05.

+ = p<0.1.

In specifications II and III, we examined the relationship between management and the quality of VMMC services, controlling for country variation, volume of outpatients and type of facility (Table 4). We did not find significant association between quality and management with the GLM model. While with the ME model, we found a significant and positive relationship between National Governance and quality.

Using the same models, GLM and Mixed-effects, we performed a backward elimination process on specifications I and II. Results are presented in Table 5.

**Table 5:** Association between management scores and efficiency and quality of HIV service delivery (*backward elimination*).

Management Variables	I: Efficiency	II: Quality
	GLM	GLM
Performance-based funding	-0.15*	
	(-0.31–-0.003)	
Non-Hospital	16*	
	(3.5–28)	
Outpatients (100s)	0.04***	
	(0.02–0.07)	
Rwanda		-25***
		(-32–-18)
Zambia		-20***
		(-29–-11)
Observations	108	96
Number of Groups		

Note: Kenya is the country of reference. GLM (Generalized Linear Models- Gaussian family), ME (Mixed-effects Models) 95% Confidence interval in parentheses.

*** = p<0.001.

* = p<0.05.

To examine the relationship between management, efficiency and quality by country, we ran efficiency and quality specifications I and II on each country sample (S5 and S6 Tables). External supervision was negatively correlated with efficiency in the case of Kenya. Community participation was positively correlated with quality in Zambia. These results need to be interpreted cautiously, as there are limitations regarding sample size. Particularly for Zambia, for which there are only fourteen observations.

## Discussion

We analyzed a sample of 108 VMMC facilities in Kenya, Rwanda, South Africa and Zambia to examine the heterogeneity of quality and efficiency, as well as the relationship between management, efficiency and quality. We found high variation in scores within and across countries in terms of both efficiency and quality. We also found substantial variation of ‘performance’, defined in terms of both efficiency and quality, across countries. Specifically, Kenyan facilities fell mostly in the quadrants characterized by high quality (quadrants I and IV), while Rwandan facilities fell principally in the low performance quadrant.

Kenyan VMMC services in our sample tended to be of high quality. This finding is consistent with previous research, [12,33] as this may be related to the relatively early (2008) rollout of VMMC services in Kenya. [27] Rwanda, in contrast, where facilities fell mostly in the low-quality quadrants (2 and 3), did not begin offering VMMC until 2010 and to date has relatively low VMMC coverage. [34,35] Maturity of VMMC programs could affect efficiency and quality, particularly in the beginning of adoption of new interventions. In a different analysis, results on the variation of unit costs of VMMC in the ORPHEA sample are consistent with this hypothesis, with more mature sites systematically showing lower unit costs.[21] However, even accounting for maturity of programs, variations persist: while Zambia and South Africa both began their VMMC programs at around the same time (in 2008), South African facilities scored high in the efficiency index but varied substantially in quality, while most of the Zambian facilities fell in the low-quality region and varied substantially in efficiency. It is possible that this could be related to South Africa’s innovative and successful partnering with private clinics to provide free VMMC services in high-need areas. [35,36]

We found evidence of statistically significant associations between management practices and efficiency.

Our findings suggest that Performance-Based Funding (PBF) is related to decreased efficiency. Literature on PBF offer useful insights on this issue. For example, De Walque *et al*. found that PBF increased the quantity of HIV testing in Rwanda.[37] Zeng *et al*., also found that the use of PBF increased the uptake of prevention of mother-to-child transmission services in Rwanda. [29] Suthar *et al*. found similar results regarding the effects of PBF on HIV service provision in Sub-Saharan African countries. In their systematic review, PBF did not increase the coverage of individual HIV testing, but they did improve take up of couple testing. [38] If the increase in quantity and the resulting gains in efficiency from economies of scale outweighs the costs of the PBF scheme, one would expect to see a positive impact on efficiency from PBF. However, if the increase of quantity is not sufficiently large to outweigh the costs of PBF, the opposite result would be expected. [37] Our results are consistent with the latter scenario. Regarding quality and National Governance, we observed a positive significant relationship in the ME model.

When we analyze the results of the analysis by country, evidence is little. This is likely because of small sample sizes.

Our study is not without limitations that should be considered when interpreting our results. As a result of the sampling strategy, the samples are not nationally representative, but rather representative of the subnational areas (states or provinces) included in the sample. With the exception of a few cases of highly violent areas, the provinces selected included those with the highest burden of HIV in each country.

Another limitation is that the sample size of facilities is relatively small—108 for the efficiency models and 96 for the quality models. Small samples were especially prevalent in country-level analyses—Zambia had only seventeen and fourteen observations for efficiency and quality models, respectively. This led to lower precision of our estimates and could explain the lack of significance of some coefficients in our models. Results from the models are thus adjusted for between-country differences in order to produce generalizable results on the relationship between management and efficiency and quality of VMMC services. In country-level analyses where sample sizes were small, results should be interpreted with caution.

Additionally, using DEA to assess efficiency has some limitations. DEA does not make comparisons to a theoretical maximum, but rather to a benchmark that is relative to the specific sample analyzed. This may limit the external validity of the results. Lastly, while we exploited to the best of our ability the survey design to create comprehensive measures of management, we recognize that there may be relevant aspects of management left unaddressed. Some facets not found here include patient flow, workflow at the facility, and target setting. These and other unmeasured managerial aspects unexplored in this analysis may impact the efficiency or quality of the VMMC services.

In addition, we used clinical vignettes to measure process quality. Clinical vignettes are measures of competence of the clinical practice. Notably, it is possible that physicians’ reported and actual practices differ. Notwithstanding potential reporting bias, studies have shown that vignettes are the most inexpensive, reliable and valid method to measure the process of care. [39,40]

Prior to the present analysis, there was little evidence on the relationship between management, efficiency, and quality in the provision of VMMC services. While observational in nature, our results are consistent with other studies that point to the importance of management practices in healthcare delivery. [17,18]

Given the need for evidence on interventions to improve efficiency without depleting quality, our results suggest that exploring the impact of management practices on quality and efficiency of health services may be a fruitful future direction. Research is required to explore if changes in management practices towards strengthening community participation and involvement in service planning and provision may catalyze an increase in the efficiency of VMMC service delivery. Another potential relationship that merits further testing is the effect of sanctions on motivation and performance of health staff. Finally, our results suggest that it is important to further explore the impact of PBF on efficiency and quality of VMMC and other services. In order to more accurately delineate the effects of these management model on HIV service delivery or healthcare services in general, however, more evidence is needed. In particular, prospective randomized trials could more accurately address the directionality of the effect, and therefore inform best practice through more specific, policy-relevant recommendations. In conclusion, this study highlights the importance to further analyze associations between management practices, quality and efficiency in VMMC service delivery—a topic that remains largely unexplored and thus ripe for future research.

## Supporting information

S1 Table Inputs and outputs used in the DEA model. (DOCX)

S2 Table Aspects included in the measurement of quality. (DOCX)

S3 Table Management variables definitions. (DOCX)

S4 Table Variable definitions. (DOCX)

S5 Table Regression between management practices and efficiency and quality of HIV service delivery by country GLM. (DOCX)

S6 Table Regression between management practices and efficiency and quality of HIV service delivery by country GLM (*backward elimination*). (DOCX)
