# Supplementary material for: The relationship between management practices and the efficiency and quality of voluntary medical male circumcision services in four African countries
Source: PLoS One. 2019 Oct 3;14(10):e0222180. doi: 10.1371/journal.pone.0222180 (PMC6776351; doi:10.1371/journal.pone.0222180)
Supplement: S1 Table — (DOCX) [file pone.0222180.s001.docx]

**S1 Table. Inputs and outputs used in the DEA model**

|  | **All sample (N=108)** | |  | **Kenya (N=33)** | |  | **Rwanda (N=32)** | |  | **South Africa (N=26)** | |  | **Zambia (N=17)** | |
| --- | --- | --- | --- | --- | --- | --- | --- | --- | --- | --- | --- | --- | --- | --- |
|  | **Mean** | **SD** |  | **Mean** | **SD** |  | **Mean** | **SD** |  | **Mean** | **SD** |  | **Mean** | **SD** |
| **INPUTS** |  |  |  |  |  |  |  | |  |  |  |  |  |  |
| Capital | $829 | $3,248 |  | $1,581 | $5,460 |  | $407 | $628 |  | $264 | $651 |  | $1,027 | $2,728 |
| Utilities | $2,256 | $6,665 |  | $260 | $390 |  | $653 | $1,542 |  | $8,182 | $11,777 |  | $83 | $91 |
| FTE Medical doctors | 0.6 | 0.9 |  | 0.8 | 0.9 |  | 0.2 | 0.7 |  | 0.9 | 1.1 |  | 0.2 | 0.4 |
| FTE Nurses | 1.8 | 2.3 |  | 1.1 | 1.3 |  | 1.6 | 2.3 |  | 3.2 | 3 |  | 1.1 | 1.4 |
| FTE Health staff | 0.8 | 1.3 |  | 1.2 | 1.1 |  | 0.4 | 0.9 |  | 1.2 | 1.8 |  | 0.4 | 0.5 |
| **OUTPUT** |  |  |  |  |  |  |  |  |  |  |  |  |  |  |
| VMMC clients | 842 | 1,379 |  | 869 | 798 |  | 342 | 392 |  | 1,665 | 2,415 |  | 470 | 533 |

***N (Number of facilities), SD (Standard deviation), FTE (Full time equivalent)**, **the inputs and outputs were estimated for a one-year period. Detailed information can be found in table S4.**
