# Supplementary material for: The relationship between management practices and the efficiency and quality of voluntary medical male circumcision services in four African countries
Source: PLoS One. 2019 Oct 3;14(10):e0222180. doi: 10.1371/journal.pone.0222180 (PMC6776351; doi:10.1371/journal.pone.0222180)
Supplement: S2 Table — (DOCX) [file pone.0222180.s002.docx]

**S2 Table. Aspects included in the measurement of Quality**

| **Question** | **Type of question** | **Items** |
| --- | --- | --- |
| If a 26-year-old man arrives for male circumcision services. As far as he knows, he is HIV-negative. The man states he wants to be circumcised. Please tell me everything you would explain to or discuss with him during pre-MC procedure evaluation and counselling | Yes/No | - Welcome to MC services at the facility |
|  |  | - General information on MC |
|  |  | - Evaluation of client knowledge of MC |
|  |  | - Discussion of how MC can reduce chances of HIV infection |
|  |  | - Risks of the MC procedure |
|  |  | - Benefits of the MC procedure |
|  |  | - Assurance that you have the freedom to choose whether or not to be circumcised |
|  |  | - Information on HIV testing in general, testing procedures, and possible test results |
|  |  | - Sexuality and gender issues |
|  |  | - Male and female sexual and reproductive health and rights |
|  |  | - Sexually transmitted infections |
|  |  | - HIV infection |
|  |  | - Correct and consistent condom use |
|  |  | - Reducing the number of sexual partners |
|  |  | - Delaying sexual debut |
|  |  | - Avoiding unprotected sex |
|  |  | - Family planning |
|  |  | - Substance use (e.g. Drugs, alcohol, tobacco) |
|  |  | - Violence, including gender-based violence |
|  |  | - Community expectations of men |
|  |  | - Goal setting and decision making |
|  |  | - Who will do the surgical procedure |
|  |  | - If the procedure is painful |
|  |  | - If the procedure will be done with an anesthetic or not |
|  |  | - How to wash the genital area and penis carefully the day of the procedure |
|  |  | - Patient consent form |
| Please tell me everything you would ask the man about his medical and personal history during his pre-MC procedure evaluation and counselling visit? | Yes/No | - Current health problems |
|  |  | - History of hemophilia or bleeding disorders |
|  |  | - Currently under treatment for anemia |
|  |  | - History of diabetes |
|  |  | - Currently under treatment for diabetes |
|  |  | - Currently under any other treatment, specify |
|  |  | - Known allergies to medications |
|  |  | - Problems with penile erections or any other concerns about sexual function |
|  |  | - Previous surgical operations |
|  |  | - Complications from previous surgical operations |
|  |  | - Has been screened, tested, or treated for any sexually transmitted infections |
|  |  | - Last time he was tested (the date or how long ago) |
|  |  | - Urethral discharge |
|  |  | - Genital sores or ulcers |
|  |  | - Painful erection |
|  |  | - Swelling of the scrotum |
|  |  | - Painful urination |
|  |  | - Difficulty in retracting foreskin |
| What physical examinations would you do to see if the man was eligible for the circumcision procedure? | Yes/No | - Urethral discharge |
|  |  | - Genital sores or ulcers |
|  |  | - Painful erection |
|  |  | - Swelling of the scrotum |
|  |  | - Painful urination |
|  |  | - Difficulty in retracting foreskin |
|  |  | - Problems with penile erection |
| Please tell me everything you would tell the man regarding what he should do or not do after the surgical procedure. | Yes/No | - What to wear after the surgical procedure |
|  |  | - How to wash the genital area and penis carefully |
|  |  | - Rest for a few days after the surgical procedure |
|  |  | - Lying down on your back takes off pressure |
|  |  | - Walk regularly after the surgical procedure |
|  |  | - Do not ride a bicycle during the days after the operation |
|  |  | - Keep the genital area dry after the operation |
|  |  | - Keep the area clean after the operation |
|  |  | - Do not use cream, ointment, or other substance on genital area after operation |
|  |  | - Do not remove bandage until told to do so |
|  |  | - Avoid sports for 4-6 weeks after procedure |
|  |  | - Avoid sex for 4-6 weeks after procedure |
|  |  | - Condom use during intercourse for at least 6 months after procedure |
|  |  | - Condom use to prevent HIV and STI infection |
| Please tell me what you would warn the man about. | Yes/No | - Continued bleeding from the wound |
|  |  | - Formation of a large blood clot under the skin near the site of operation |
|  |  | - Continuous or excessive pain |
|  |  | - Swelling |
|  |  | - Discharge of fluid or pus, which can indicate infection |
| Other Questions | Yes/No | - Would you give the man a contact number before sending him home? |
|  |  | - Would you give the man information on where to go in case of an emergency? |
|  |  | - After post-MC counselling, would you refer the man for further HIV testing and counselling? |
|  |  | - After post-MC counselling, would you schedule a follow-up appointment for the man? |
