# Supplementary material for: The relationship between management practices and the efficiency and quality of voluntary medical male circumcision services in four African countries
Source: PLoS One. 2019 Oct 3;14(10):e0222180. doi: 10.1371/journal.pone.0222180 (PMC6776351; doi:10.1371/journal.pone.0222180)
Supplement: S3 Table — (DOCX) [file pone.0222180.s003.docx]

**S3 Table. Management variables definitions**

| **Variable name** | **Description** | **Questions** |
| --- | --- | --- |
| Performance-based funding | Additive score of all types of incentives applied at the facility level. | Is funding directly linked or based on:  1) Facility performance  2) Number of clients served  3) Number of services delivered  4) Amount of inputs used  5) Quality of service  6) Level of supplies (*i.e.* no stockouts |
| Sanctions | Additive score of all types of sanctions applied at the facility level. | Does the facility warn or apply sanctions for:  1) Unprofessional behaviour  2) Not performing responsibilities  3) Absence without cause  4) Persistent tardiness  5) Persistent absenteeism  6) Impoliteness to patients  7) Impoliteness to supervisors  8) Persistent rudeness  9) Receiving many patient complaints  10) Theft |
| External supervision | Additive score of all types of aspects for which the facility received supervision by a higher-level facility during the costing year. | Since 2011, has a higher level facility provided supervision to this facility for [. . . ]?   1. Admin 2. Financial management 3. M&E 4. Supply chain management 5. Capacity management |
| Community participation | Additive score of all types of aspects in which the community council was involved in the facility during the costing year. | 1) Does a community advisory council exist for this facility?  Since 2011, has the council …  2) Bought items or awarded personnel bonuses from available funds  3) Communicated patient complaints or expressions of gratitude to the facility  4) Monitored the delivery of HIV-related drugs to the facility  5) Monitored the delivery of HIV-related supplies to the facility  6) Participated in discussions about priorities at the facility  7) Participated in discussions about allocations for the various services  8) Liaise with higher levels of government about services provided  9) Does the community have a role in monitoring and providing feedback to improve the performance of the facility?  10) Does this facility involve community groups in providing health service provision to the community? |
| National governance | Additive score of all national agencies involved in budgetary and expenditure decisions at the facility during the costing year. | Are budgetary decisions at the facility made by:   1. National Agency for the Control of AIDS 2. National health authorities 3. National government   Are expenditure decisions at the facility made by:   1. National Agency for the Control of AIDS 2. National health authorities 3. National government |
| Municipal governance | Additive score of all municipal agencies involved in budgetary and expenditure decisions at the facility during the costing year. | Are budgetary decisions at the facility made by:   1. Local or municipal authorities 2. Local or municipal health authorities   Are expenditure decisions at the facility made by:   1. Local of municipal authorities 2. Local of municipal health authorities |
