# Supplementary material for: The relationship between management practices and the efficiency and quality of voluntary medical male circumcision services in four African countries
Source: PLoS One. 2019 Oct 3;14(10):e0222180. doi: 10.1371/journal.pone.0222180 (PMC6776351; doi:10.1371/journal.pone.0222180)
Supplement: S4 Table — (DOCX) [file pone.0222180.s004.docx]

**S4 Table. Variable definitions**

| **Variable name** | **Type** | **Description** |
| --- | --- | --- |
| *Inputs* | | |
| Capital | Continuous | Cost of capital are expressed in 2013 US dollars. Capital included annual cost of equipment and vehicles assuming a useful life of ten years. As capital is shared between several interventions, the MC capital cost was obtained by multiplying the total annualized capital cost by a client-based weight, ie. the total number of VMMCs performed at the facility divided by the total number of outpatient clients at the facility in the costing year |
| Utilities | Continuous | Cost of utilities are expressed in 2013 US dollars. Utilities included water and electricity services. The total cost of utilities was calculated by adding the monthly cost of water and electricity services over the costing year. As the facility used these services to provide other interventions, the cost share for MC was obtained using a client-based weight, as above (total number of VMMCs performed at the facility divided by the total number of clients at the facility in the costing year) |
| FTE Medical Doctors | Continuous | Number of full time-equivalent medical doctors that participate in the provision of VMMC |
| FTE Nurses | Continuous | Number of full time-equivalent general nurses that participate in the provision of VMMC |
| FTE of other Health Staff | Continuous | Number of full time-equivalent health staff that participate in the provision of VMMC |
| *Outputs* | | |
| VMMC Clients | Continuous | Number of VMMC annual clients |
| *Efficiency* | | |
| Efficiency score | Continuous | Efficiency score estimated through DEA (0 – 100) |
| *Facility characteristics* | | |
| Type of facility | Binary | Indicator variable for level of service provision, dichotomized into hospitals (reference category) and primary care clinics |
| *Location* | | |
| Countries | Categorical | Indicator variables for countries, including Kenya (reference category), Rwanda, South Africa, Zambia |
