# Supplementary material for: The relationship between management practices and the efficiency and quality of voluntary medical male circumcision services in four African countries
Source: PLoS One. 2019 Oct 3;14(10):e0222180. doi: 10.1371/journal.pone.0222180 (PMC6776351; doi:10.1371/journal.pone.0222180)
Supplement: S5 Table — (DOCX) [file pone.0222180.s005.docx]

**S5 Table. Regression between management practices and efficiency and quality of HIV service delivery by country GLM**

|  | Specification I | Specification II | Specification III | Specification IV | Specification V | Specification VI | Specification VII | Specification VIII |
| --- | --- | --- | --- | --- | --- | --- | --- | --- |
|  | KENYA | | RWANDA | | SOUTH AFRICA | | ZAMBIA | |
| **Management Variables** | **Y=Efficiency score** | **Y=Quality score** | **Y=Efficiency score** | **Y=Quality score** | **Y=Efficiency score** | **Y=Quality score** | **Y=Efficiency score** | **Y=Quality score** |
| Performance-based funding | -0.06 | 0.04 | 0.2 | 0.08 | -0.53+ | 0.2 | 0.1 | -0.13 |
|  | (-0.41 - 0.27) | (-0.11 - 0.20) | (-0.47 - 0.85) | (-0.22 - 0.38) | (-1.0 - 0.004) | (-0.16 - 0.56) | (-0.54 - 0.77) | (-0.36 - 0.09) |
| Sanctions | 0.3 | -0.14+ | -0.41 | -0.04 | -1.4 | -0.91 | -3.18 | -1.9 |
|  | (-0.09 - 0.68) | (-0.31 - 0.02) | (-1.0 - 0.21) | (-0.33 - 0.24) | (-3.8 - 1.0) | (-2.4 - 0.6) | (-7.7 - 1.4) | (-4.4 - 0.6) |
| External supervision | -0.42* | 0.15+ | 0.1 | -0.005 | 0.06 | 0.05 | 0.53 | -0.3 |
|  | (-0.82 - -0.03) | (-0.02 - 0.32) | (-0.37 - 0.58) | (-0.22 - 0.21) | (-0.34 - 0.48) | (-0.20 - 0.32) | (-0.92 - 2) | (-0.79 - 0.18) |
| Community participation | 0.3 | -0.007 | 0.25 | 0.04 | 0.06 | -0.5 | 0.12 | 0.53+ |
|  | (-0.18 - 0.77) | (-0.22 - 0.21) | (-0.34 - 0.85) | (-0.23 - 0.32) | (-0.96 - 1.1) | (-1.2 - 0.15) | (-1.3 - 1.6) | (-0.03 - 1.1) |
| National governance | 0.2 | 0.12 | 0.21 | 0.06 | 0.01 | 0.17 | 0.78 | 0.41 |
|  | (-0.25 - 0.66) | (-0.06 - 0.31) | (-0.53 - 0.97) | (-0.28 - 0.41) | (-0.4 - 0.42) | (-0.09 - 0.43) | (-0.62 - 2.2) | (-0.43 - 1.3) |
| Municipal governance | 0.1 | 0.02 | -0.001 | -0.15 | -0.21 | 0.5 | -0.97 | -0.48 |
|  | (-0.20 - 0.42) | (-0.12 - 0.17) | (-0.58 - 0.58) | (-0.43 - 0.12) | (-1.2 - 0.82) | (-0.15 - 1.2) | (-2.4 - 0.41) | (-1.3 - 0.35) |
| Outpatients (100s) | -0.07 | 0.01 | 0.1* | -0.01 | 0.003 | 0.01 | 0.16** | 0.01 |
|  | (-0.21 - 0.07) | (-0.05 - 0.08) | (0.01 - 0.19) | (-0.05 - 0.02) | (-0.04 - 0.05) | (-0.01 - 0.04) | (0.05 - 0.28) | (-0.03 - 0.05) |
| Non-Hospital | 11 | 8.5 | -1.4 | -2.2 | 10 | 4.7 | 8.2 | 3.8 |
|  | (-15 - 38) | (-3.1 - 20) | (-43 - 40) | (-22 - 18) | (-20 - 41) | (-16 - 25) | (-35 - 52) | (-11 - 19) |
| Observations | 33 | 28 | 32 | 29 | 26 | 25 | 17 | 14 |

95% Confidence interval in parentheses.

***=p<0.001, **=p<0.01, *=p<0.05, +=p<0.1

GLM (Generalized Linear Models)
