# Supplementary material for: The relationship between management practices and the efficiency and quality of voluntary medical male circumcision services in four African countries
Source: PLoS One. 2019 Oct 3;14(10):e0222180. doi: 10.1371/journal.pone.0222180 (PMC6776351; doi:10.1371/journal.pone.0222180)
Supplement: S6 Table — (DOCX) [file pone.0222180.s006.docx]

**S6 Table. Regression between management practices and efficiency and quality of HIV service delivery by country (*backward elimination*)**

|  | Specification I | Specification II | Specification III | Specification IV | Specification V | Specification VI | Specification VII | Specification VIII |
| --- | --- | --- | --- | --- | --- | --- | --- | --- |
|  | KENYA | | RWANDA | | SOUTH AFRICA | | ZAMBIA | |
| **Management Variables** | **Y=Efficiency score** | **Y=Quality score** | **Y=Efficiency score** | **Y=Quality score** | **Y=Efficiency score** | **Y=Quality score** | **Y=Efficiency score** | **Y=Quality score** |
| Performance-based funding |  |  |  |  | -0.37+ | 0.27+ |  |  |
|  |  |  |  |  | (-0.75 - 0.005) | (-0.01 - 0.55) |  |  |
| Sanctions |  | -0.12+ | -0.43* |  |  |  |  |  |
|  |  | (-0.27 - 0.02) | (-0.80 - -0.05) |  |  |  |  |  |
| External supervision | -0.31* | 0.12+ |  |  |  |  |  |  |
|  | (-0.59 - -0.03) | (-0.006 - 0.26) |  |  |  |  |  |  |
| Community participation |  |  |  |  |  | -0.59* |  |  |
|  |  |  |  |  |  | (-1.1 - -0.03) |  |  |
| National governance |  | 0.15+ |  |  |  |  |  |  |
|  |  | (-0.01 - 0.31) |  |  |  |  |  |  |
| Municipal governance |  |  |  | -0.11+ |  | 0.59* |  |  |
|  |  |  |  | (-0.24 - 0.007) |  | (0.01 - 1.2) |  |  |
| Outpatients (100s) |  |  | 0.12*** |  |  |  | 0.11*** |  |
|  |  |  | (0.05 - 0.19) |  |  |  | (0.04 - 0.17) |  |
| Observations | 33 | 28 | 32 | 29 | 26 | 25 | 17 | 14 |

95% Confidence interval in parentheses.

***=p<0.001, **=p<0.01, *=p<0.05, +=p<0.1

GLM (Generalized Linear Models)
